# Phenothiazinium Dyes Are Active against* Trypanosoma cruzi* In Vitro

**DOI:** 10.1155/2019/8301569

**Published:** 2019-07-04

**Authors:** Gisele Bulhões Portapilla, Luiz Miguel Pereira, Cássia Mariana Bronzon da Costa, Maiara Voltarelli Providello, Pedro Alexandre Sampaio Oliveira, Amanda Goulart, Naira Ferreira Anchieta, Mark Wainwright, Gilberto Úbida Leite Braga, Sérgio de Albuquerque

**Affiliations:** ^1^Faculdade de Ciências Farmacêuticas de Ribeirão Preto, Universidade de São Paulo, Av do Café, sn/n, 14040-903 Ribeirão Preto, SP, Brazil; ^2^School of Pharmacy and Biomolecular Sciences, Liverpool John Moores University, Liverpool L3 3AF, UK

## Abstract

Chagas disease is a tropical illness caused by the protozoan* Trypanosoma cruzi*. The disease affects populations of the Americas and has been spread to other continents due to the migration process. The disease is partially controlled by two drugs, Benznidazole and Nifurtimox. These molecules are active in the acute phase of the infection but are usually ineffective during the symptomatic chronic phase. Several research groups have developed novel candidates to control Chagas disease; however, no novel commercial formulation is available. In this article, we described the anti-*T. cruzi *effects of phenothiazinium dyes in amastigote and trypomastigote forms of the parasite. Methylene Blue, New Methylene Blue, Toluidine Blue O, and 1,9-Dimethyl Methylene Blue inhibited the parasite proliferation at nanomolar concentrations and also demonstrated low toxicity in host cells. Moreover, combinations of phenothiazinium dyes indicated a synergic pattern against amastigotes compared to the Benznidazole counterparts. Phenothiazinium dyes levels of reactive oxygen species (ROS) and decreased the mitochondrial potential in trypomastigotes, indicating the mechanism of action of the dyes in* T. cruzi*. Our article offers a basis for future strategies for the control of Chagas disease using low-cost formulations, an important point for endemic underdeveloped regions.

## 1. Introduction

*Trypanosoma cruzi *is the etiologic agent of the Chagas disease, endemic in the Americas. Chagas disease afflicts ~ 6 million to 7 million people and nowadays has spread to North America, Europe, Asia, and Oceania due to migration. The disease has two distinct phases; the acute phase is asymptomatic or causes unspecific symptoms. The chronic phase, when symptomatic, may lead to cardiac and/or digestive degeneration [1].

Despite the effort of several groups to develop novel therapies [2], there are only two commercial drugs for Chagas disease control, Benznidazole and Nifurtimox [3]. Moreover, several cases of treatment failure have been described [4], demanding new active and safe drugs. In this article, we investigated the anti-*T. cruzi* effect of phenothiazinium dyes, a low-cost family of drugs with potential against malaria [5]. Methylene Blue (MB), the most used phenothiazinium dye, has been applied against malaria since the XIX century, representing the first report of treatment with a synthetic molecule [6]. Due to the reversible side effects (green sclera and urine), MB was replaced by alternative antimalarial molecules, such as quinine, artemisinin, and chloroquine [7]. Nowadays MB has been revived for control of malaria, cancer, cyanide poisoning, and methaemoglobinaemia and recently in the treatment of patients with Alzheimer's disease [8].

In this study, we evaluated the potential of the phenothiazinium dyes for the control of* T. cruzi in vitro*. We also determined several synergic formulations composed by phenothiazinium dyes and/or Benznidazole. ROS production and mitochondrial activity in treated cultures were assayed by flow cytometry, in order to investigate some aspects of the mechanism of action of phenothiazinium dyes in* T. cruzi*. The screening of low-cost compounds is a key factor for the control of the Chagas disease in endemic regions, most of them being in a delicate economic state.

## 2. Material and Methods

### 2.1. *T. cruzi* Culture

*T. cruzi *(Tulahuen strain) was maintained in cultures of LLCMK2 cells and cultivated in RMPI supplemented with 10% fetal bovine serum (FBS) at 37°C, 5% CO_2_. The parasites used in proliferation and flow cytometry assays were genetically modified to express the enzyme *β*-galactosidase (*T. cruzi*-LacZ [9]). To illustrate the inhibition of parasite proliferation, a* T. cruzi* expressing GFP (G-GFP strain [10]) was used.

### 2.2. Compounds

Methylene Blue (MB), New Methylene Blue (NMB), Toluidine Blue O (TBO), and 1,9–Dimethyl Methylene Blue (DMMB) were purchased from Sigma-Aldrich and diluted in PBS at 5 mg/ml. Benznidazole (BZ) and Menadione (MN) was purchased from Sigma-Aldrich and diluted at 5 mg/ml in dimethyl sulfoxide (DMSO).

### 2.3. Amastigote Growth Inhibition Assay

LLCMK2 cells were distributed in 96-well plates (5×10^4^ cells/ml) and infected with* T. cruzi*-LacZ at a multiplicity of infection (MOI) of 10:1 (5×10^5^ cells/ml). The cultures were incubated for 48 h, 37°C, and 5% CO_2_ and washed with phosphate buffered saline (PBS). Serial dilutions of compounds (starting from 10 *μ*M) in RPMI were added to the cultures in duplicate and incubated for 72 h, 37°C,and 5% CO_2_. For the drug combination assay, fractioned dilutions (2 × IC_50_, 1.6 × IC_50_, 1.3 × IC_50_, 0.7 × IC_50_, 0.5 × IC_50_, and 0.2 × IC_50_) of single and combined compounds [11] were added to the infected cultures and incubated under the same conditions. We considered 8 *μ*M, 1 *μ*M, 0.05 *μ*M, 0.7 *μ*M, and 0.04 *μ*M as 2 × IC_50_ for BZ, MB, NMB, TBO, and DMMB, respectively. After incubation, the media were removed and followed by the reaction with chlorophenol red-*β*-D-galactopyranoside (CPRG) buffer (200 *μ*M CPRG, 2% Trion X-100, and 50 mM MgCl_2_ in PBS) for 4 h, 37°C. The plates were read at 570 nm in ELISA reader (Synergy™ H1, Biotek). Three independent assays were performed.

### 2.4. Fluorescence Microscopy

LLCMK2 cells (2 × 10^3^ cells per well) were cultivated in 96-well, black, flat-bottomed plates for 6 h, 37°C, and 5% CO_2_. The plate was washed with PBS and the cells were infected with* T. cruzi* (G-GFP strain) at a multiplicity of infection (MOI) of 5:1 (1×10^5^ cells/ml). The cultures were incubated for 24 h, 37°C, 5% CO_2,_ and the wells washed 3 times with PBS in order to remove noninternalized parasites. Subsequently, IC_50_ concentrations of MB, NMB, TBO, DMMB, and BZ were added to the cultures and the plates were incubated for 72 h, 37°C, and 5% CO_2_. Infected and nontreated and noninfected cells were used as negative controls and positive controls, respectively. After the incubation, the plates were fixed with 4% paraformaldehyde for 20 minutes, followed by washing with PBS. The nucleus of host cells was stained with 0.25 *μ*M 4′,6-diamidine-2′-phenylindole dihydrochloride (DAPI) (Ex/Em = 340/488 nm) and the GFP* T. cruzi* parasites were detected by the green florescence (Ex/Em = 488/510 nm). The images were acquired in an Image Xpress Micro XLS Widefield High-Content Analysis System from Molecular Devices. The system also calculated the percentage of inhibition compared to the nontreated control. This was done using the number of intracellular amastigotes in twenty-five images obtained for each well.

### 2.5. Cytotoxicity

The cytotoxic effects of the phenothiazinium dyes in LLCMK2 cells was measured by Thiazolyl Blue Tetrazolium Bromide (MTT) assay, as described in [12]. A suspension of LLCMK2 cells (5 × 10^4^/ml) in RPMI supplemented with 10% FBS was distributed in 96-well plates and cultivated (37°C, 5% CO_2_) to the confluence. The media were discarded and serial dilutions of the compounds were added (starting from 100 *μ*M) in duplicate. The cultures were incubated for 72 h, 37°C, and 5% CO_2_. For the measurement of the cellular esterase activity, the media were carefully removed and an MTT solution (500 *μ*g/ml MTT in PBS) was added. The reaction was performed for 4 h at 37°C, 5% CO_2_. After the reaction, the MTT solution was discarded and the formazan crystals solubilized with DMSO. The absorbance was measured in an ELISA reader (Synergy™ H1, Biotek) at 570 nm.

### 2.6. ROS Measurement and Evaluation of Mitochondrial Membrane Potential Status

LLCMK2 cells in 75 cm^2^ flasks were cultivated to confluence and were infected with 1 × 10^7^* T. cruzi *trypomastigotes. The cultures were cultivated for 6 days when free trypomastigotes were observed in the supernatant. The infected cells were centrifuged for 10 min, 3000 g, and washed with PBS and suspended in trypsin to separate cells and parasites. The cultures were distributed in 1.5 ml microtubes (~ 1 × 10^6^ trypomastigotes/tube) and incubated with 4 × IC_50_ of compounds at 37°C, 5% CO_2_ for 2 h. Menadione (MN), a classic inducer of cellular oxidative stress [13], was used as the positive control of ROS production as previously described in [14]. After treatment, the cultures were centrifuged for 10 minutes, 3000 g, and washed with PBS and suspended in trypsin. The ROS measurement was performed after incubation with 5 *μ*M 2,7–dichlorofluorescin diacetate (DCFDA) for 15 minutes at room temperature in the dark. Similarly, the mitochondrial membrane potential was determined after incubation with 5 *μ*M JC-1 under the same conditions. JC-1 is a cationic dye that accumulates in active mitochondria. In low concentrations, the dye exhibits a green fluorescence. In polarized mitochondria, the dye forms J-aggregates, emitting red fluorescence. The cultures were washed with PBS and analyzed in a BD FACS-Canto (BD Biosciences) flow cytometer with the FACSDiva (BD) 6.1.3 software. Due to the size, host cells (infected and noninfected) and trypomastigotes were separately analyzed by flow cytometry [11]. The median of fluorescence of oxidized DCF and the populations with active mitochondria were measured with excitation/emission at 488 nm/529 nm and 488 nm/529 nm, respectively. The percentage of DCF fluorescence or cells with polarized mitochondria was calculated in relation to the respective nontreated controls.

### 2.7. Statistical Analysis

The percentages of proliferation and cytotoxicity were calculated using the mean absorbance of the compound-free control and the absorbance of each treatment. The IC_50_ and the combination index (CI) were achieved using Compusyn software (http://www.combosyn.com/) and the selective index (CC_50_/IC_50_) was also determined. From CI values, synergistic (CI < 1), and antagonistic (CI > 1) interactions were identified [15]. For oxidative stress and mitochondrial activity, data were analyzed by one-way ANOVA followed by a Dunnett's post hoc test (compared to nontreated groups) on GraphPad 5.0 software.

## 3. Results and Discussion

### 3.1. Phenothiazinium Dyes Inhibit the Amastigote Proliferation at Low Concentrations

**Figure 1:**
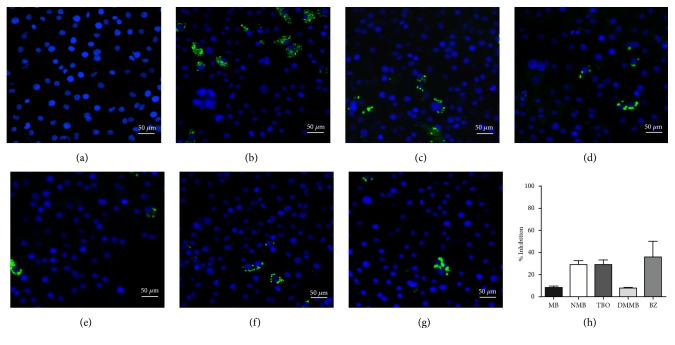
*Detection of T. cruzi in cultures treated with phenothiazine dyes or Benznidazole. T. cruzi* was added to LLCMK2 cell monolayers and cultivated for 24 h, 37°C, 5% CO_2_. After washing with PBS, the* T. cruzi* cultures were incubated with the IC_50_ concentrations of MB (0.45 *μ*M), NMB (0.09 *μ*M), TBO (0.28 *μ*M), DMMB (0.08 *μ*M), and BZ (3.5 *μ*M) for 72 hours, 37°C, and 5% CO_2_. The controls were composed by noninfected (negative control) or infected and nontreated cells (positive control) and cultivated at the same conditions. After treatment, the cultures were fixed with 4% paraformaldehyde for 20 minutes and washed with PBS and then the cell nucleus was stained with DAPI (Ex/Em = 340/488 nm). The parasites were detected by the emission of green fluorescence (Ex/Em = 488/510 nm). All figures were captured and analyzed in an Image Xpress Micro XLS Widefield High-Content Analysis System from Molecular Devices. The system also calculated the number of amastigotes in twenty-five images/well (x400). (a) Noninfected control. (b) Infected and nontreated control. (c), (d), (e), (f), and (g) Infected cultures treated with MB, NMB, TBO, DMMB,and BZ, respectively. (h) Percentage of inhibition compared to the nontreated control.

The phenothiazinium dyes inhibited the amastigote proliferation at low concentrations. The MB, NMB, TBO, and DMMB IC_50_ concentrations were lower compared to BZ (3.41 *μ*M), reaching 0.44 *μ*M, 0.09 *μ*M, 0.27 *μ*M, and 0.08 *μ*M, respectively (Table 1). Moreover, a similar inhibition pattern between BZ and phenothiazinium dyes was observed using high-content screening analysis by amastigotes counting (Figure 1). At IC_50_ concentrations (Table 1) all tested compounds partially inhibited parasite proliferation (Figures 1(c), 1(d), 1(e), 1(f), 1(g), and 1(h)), compared to the nontreated control (Figure 1(b)). However, the phenothiazinium dyes demonstrated higher cytotoxicity (lower CC_50_) in relation to BZ (> 200 *μ*M) wherein MB, NMB, TBO, and DMMB inhibited the host cell reductases at 16.82 *μ*M, 4.19 *μ*M, 7.93 *μ*M, and 4.46 *μ*M, respectively. Despite the relative higher host cell toxicity, the selective index (SI) was above 25, which is adequate compared to the index recommended for candidate drugs for Chagas disease control. In drug screening procedures against* T. cruzi*, an SI > 10 is recommended for candidates with potential for use in* in vivo* and clinical assays [16, 17]. Moreover, MB has been successfully applied against malaria and to treat methemoglobinemia with few cases of toxicity [18, 19], reinforcing its potential use as anti-*T. cruzi* compound in further assays.

### 3.2. Phenothiazinium Combinations Are Synergic against Amastigotes* In Vitro*

**Figure 2:**
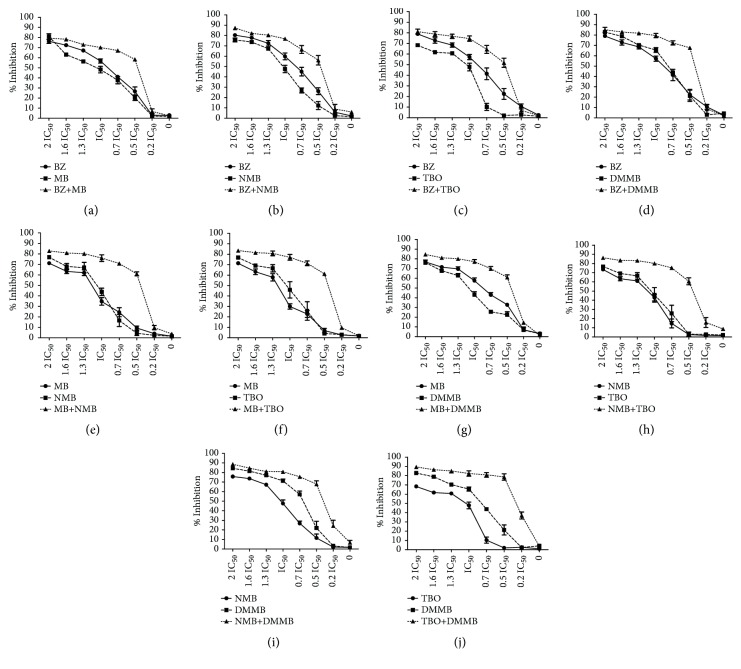
*Inhibitory activity of phenothiazinium dyes combinations against T. cruzi. *Amastigotes were incubated with seven dilutions (2 × IC_50_, 1.6 × IC_50_, 1.3 × IC_50_, 0.7 × IC_50_, 0.5 × IC_50_, and 0.2 × IC_50_) of MB + BZ (a), NMB + BZ (b), TBO + BZ (c), DMMB + BZ (d), MB + NMB (e), MB + TBO (f), MB + DMMB (g), NMB + TBO (h), NMB + DMMB (i), TBO + DMMB (j), and the proliferation measured after CPRG assay. As references, isolated compounds of each combination were evaluated concomitantly in the same plate. The inhibition percentage was calculated in comparison to the nontreated group and the IC_50_ and CI values were achieved using Compusyn software.

The combination of compounds has an important potential for the control of the Chagas disease. Several combinations of BZ with Itraconazole, E1224 (ravuconazole prodrug), and ketoconazole have demonstrated improved anti-*T. cruzi* pattern compared to the single treatments [20–22]. The main aim of combinations with BZ is the decrease of dosages allied to the alleviation of side effects, which allow longer-term treatments. However, the combinations of phenothiazinium dyes with BZ resulted in an antagonist effect (CI > 1), despite the elevation of the parasite inhibition, mostly at lower concentrations (Figures 2(a), 2(b), 2(c), 2(d), and Table 2). However, the combinations among phenothiazinium dyes were synergic against amastigotes (except MB + DMMB), improving the parasite inhibition in all concentrations analyzed (Figures 2(e), 2(f), 2(g), 2(h), 2(i), and 2(j)). The CI of MB + NMB, MB + TBO, MB + DMMB, NMB + TBO, NMB + DMMB, and TBO + DMMB were 0.90, 0.89, 1.05, 0.74, 0.80, and 0.42, respectively (Table 2). The CI values probably indicate some differences in mechanisms of action among the phenothiazinium dyes. For example, the combinations containing TBO demonstrated the lowest CI values, probably due to low structure similarity with other dyes. In contrast, MB and DMMB are structurally similar, which reflected in an antagonist pattern when combined (Table 2). However, TBO demonstrated a high IC_50_ (compared to NMB or DMMB), which may affect negatively further* in vivo* treatments. Therefore, a wide and complex strategy for* in vivo* procedures is demanded, once there are multiple combinations and treatment regimens (acute, chronic, and pregnant models) to test.

### 3.3. Phenothiazinium Dyes Increase ROS in Treated Cells and Trypomastigotes

**Figure 3:**
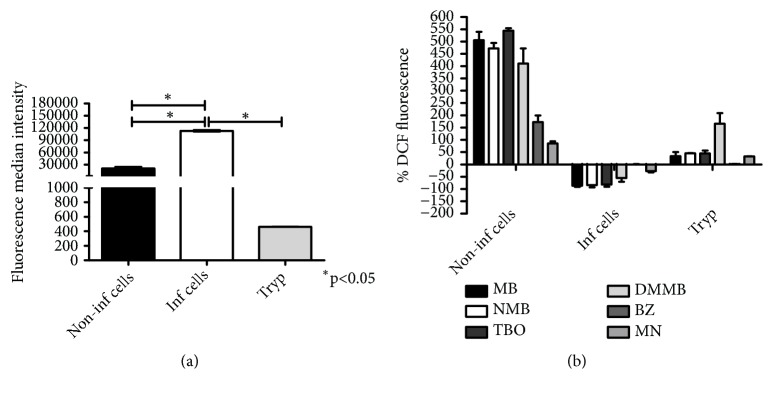
*ROS detection in T. cruzi cultures treated with phenothiazinium dyes. T. cruzi* cultures were incubated with trypsin and treated with 4 × IC_50_ MB, NMB, TBO, DMMB, BZ, and MN for 2 hours, 37°C, and 5% CO_2_. The cultures were washed with PBS and incubated with 5 *μ*M DCFDA for 15 minutes in the dark. The samples were analyzed in a flow cytometer and the median intensity of fluorescence used for ROS measurement. The control was composed of noninfected LLCMK2 cells processed under the same conditions. The percentage of fluorescence (% DFC fluorescence) was calculated in relation to the nontreated control. (a) Median intensity of fluorescence from nontreated LLCMK2 cells and trypomastigotes. (b) Percentage of DCF fluorescence from LLCMK2 cells (infected and noninfected) and trypomastigotes treated with phenothiazinium dyes, BZ, and MN.

The literature is contradictory regarding the role of ROS produced by the host cell during* T. cruzi *infection. Several studies have implicated the host cell respiratory burst in controlling the infection [23, 24]. Others have reported a positive correlation between ROS production by the host and parasite proliferation [25, 26]. Also, when a sublethal hydrogen peroxide concentration was added to a* T. cruzi* infected culture, parasite proliferation increased [27]. Our results show increased ROS production in* T. cruzi* infected cells (untreated) when compared to noninfected cells (Figure 3(a)), corroborating an effect also observed for proliferating bacteria [28], fungi [29], viruses [30, 31], and* Leishmania* [32]. Despite the efficient antioxidant machinery [33], we observed increased ROS production in* T. cruzi* trypomastigotes after treatment with phenothiazinium dyes (Figure 3(b)). This effect was similar to the one observed for MN a classic inducer of oxidative stress [13]. On the other hand, no ROS production was detected in trypomastigotes treated with BZ. Although BZ induces the production of free radicals and electrophilic metabolites after the reduction of its nitro group, no ROS is produced in treated parasites [34]. We have observed that treatment with phenothiazinium dyes (and to a lesser extent BZ and MN) increased ROS production in noninfected cells (Figure 3(b)). In contrast, treating* T. cruzi* infected cells with phenothiazinium dyes decreased ROS production (Figure 3(b)). Indeed, Methylene Blue scavenges ROS in several biological models such as skin and HT-22 cells, elevating oxygen consumption and mitochondrial activity [35–37]. Because* T. cruzi* proliferation is stimulated by ROS production [27], we speculate that scavenging of such ROS by phenothiazinium dyes impairs parasite proliferation. However, for the complete elucidation of the roles of phenothiazinium dyes in ROS scavenging, complementary assays will be required in further studies. As an example, the ability of phenothiazinium dyes to scavenge ROS may be compared to that of Trolox (6-Hydroxy-2,5,7,8-tetramethylchromane-2-carboxylic acid), a well-established ROS scavenger [38, 39].

### 3.4. Mitochondria of Trypomastigotes Are Sensitive to Phenothiazinium Dyes

**Figure 4:**
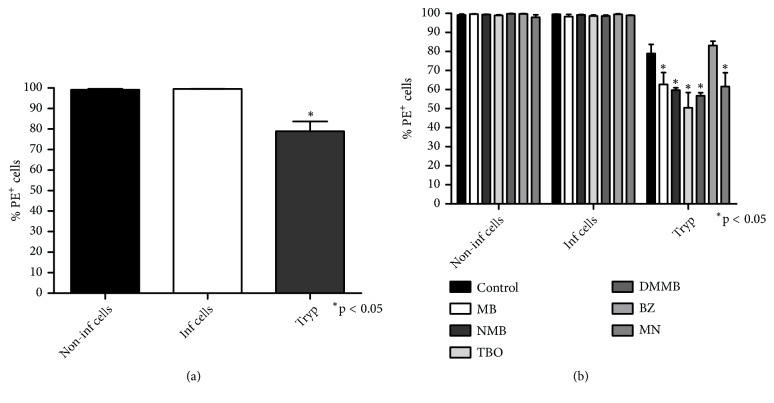
*Mitochondrial activity of T. cruzi cultures treated with phenothiazinium dyes. T. cruzi* cultures were incubated with trypsin and treated with 4 × IC_50_ MB, NMB, TBO, DMMB, BZ, and MN for 2 hours, 37°C, and 5% CO_2_. The cultures were washed with PBS and incubated with 5 *μ*M JC-1 for 15 minutes in the dark. The samples were analyzed in a flow cytometer and the positive red fluorescent cells (PE^+^ cells) determined. The control was composed of noninfected LLCMK2 cells processed under the same conditions. The percentage of positive red fluorescent cells (% PE^+^ cells) was calculated in relation to the nontreated control. (a) Positive red fluorescent cells from nontreated LLCMK2 cells and trypomastigotes. (b) Percentage of positive red fluorescent cells from LLCMK2 cells (infected and noninfected) and trypomastigotes treated with phenothiazinium dyes, BZ, and MN.

All noninfected and infected LLCMK2 cells are positive to JC-1 accumulation, indicating the presence of active mitochondria. However, 22% of trypomastigotes were negative to mitochondrial polarization (Figure 4(a)). Moreover, phenothiazinium dyes decreased mitochondrial membrane potential in trypomastigotes, whereas the infected and noninfected cells were unaffected by incubation with the compounds (Figure 4(b)). Also, BZ and MN displayed distinct effects on the mitochondrial membrane potential of trypomastigotes. The former did not affect mitochondria whereas the latter reduced the membrane potential (Figure 4(b)). It was previously observed that BZ elevates free radical production and causes DNA damage without affecting mitochondria [40]. The effect of phenothiazinium dyes on trypomastigotes was similar to that observed for MN (Figure 4(b)), a classic mitochondrial inhibitor [41]. Thus, the increment of ROS observed in trypomastigotes under treatment with dyes may be a consequence of mitochondrial depolarization. Moreover, decreased mitochondrial activity may be also related to the ROS impairment production in infected cells treated with phenothiazinium dyes. Drugs that alter the mitochondrial activity usually lead to loss of parasite ATP production and induction of apoptosis [42]. For example, MB improves mitochondrial respiration in cells [35, 43], decreasing ROS in cardiac tissue from diabetic rats [44].

We have observed that active and synergic combinations of phenothiazinium dyes are effective in killing* T. cruzi*. Our results also indicate the potential of phenothiazinium dyes as candidates for the control of* T. cruzi*. We speculate that phenothiazinium dyes scavenge ROS during* T. cruzi* infection thus reducing parasite proliferation. However, future assays will be necessary to elucidate the mechanism. Furthermore, the induction of apoptosis by disruption of mitochondrial polarity should also be considered as a potential mechanism operating in conjunction with the ROS scavenging by phenothiazinium dyes. The present article opens the perspective for the development of low-cost and active anti-*T. cruzi* molecules, pivotal characteristics for the control of Chagas disease in low-middle-income endemic regions.

## Figures and Tables

**Table 1:** *In vitro IC*_50_ *and toxicity of MB, NMB, TBO, and DMMB to T. cruzi amastigotes and LLCMK2 cells.* The molecular weight (MW), IC_50_, CC_50_, and selectivity index (SI) were calculated for MB, NMB, TBO, DMMB, and BZ on *T. cruzi* and LLCMK2 cells. Amastigotes or LLCMK2 cells were incubated for 72 hours, at 37°C, with 5% CO_2_, and the proliferation (amastigotes) or toxicity (LLCMK2 cells) was measured after CRPG or MTT assays, respectively. The percentage of inhibition was calculated in comparison to the nontreated controls in three independent assays.

Compound		MW	IC_50_ *μ*M	CC_50_ *μ*M	SI
MB	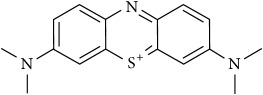	319.85	0.44 ± 0.15	16.82 ± 1.18	37.40
NMB	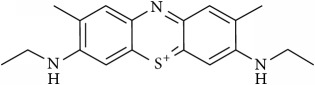	347.91	0.09 ± 0.01	4.19 ± 1.30	44.71
TBO	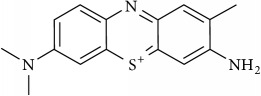	305.83	0.27 ± 0.11	7.93 ± 5.22	28.61
DMMB	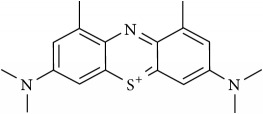	416.05	0.08 ± 0.02	4.46 ± 0.23	51.26
BZ	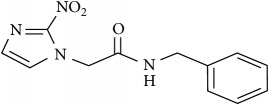	260.25	3.41 ± 0.38	> 200	> 58.65

**Table 2:** *Values of inhibitory concentrations (IC*_50_ *) and Combinatory Index of the phenothiazinium combinations.* The IC_50_ concentrations of the dyes alone and in combinations were calculated using the Compusyn software. The software was also used for the determination of the CI between the combined compounds.

Combination (compound 1 + 2)	IC_50_ (compound 1/*μ*M)	IC_50_ (compound 2/*μ*M)	IC_50_ (combination compound 1/*μ*M)	IC_50_ (combination compound 2/*μ*M)	CI
BZ + MB	3.315	0.477	2.262	0.291	1.29
BZ + NMB	2.937	0.100	2.091	0.056	1.26
BZ + TBO	3.024	0.380	2.259	0.178	1.40
BZ + DMMB	3.015	0.078	1.943	0.050	1.28
MB + NMB	0.560	0.112	0.247	0.051	0.90
MB + TBO	0.588	0.322	0.250	0.150	0.89
MB + DMMB	0.391	0.095	0.223	0.044	1.05
NMB + TBO	0.122	0.322	0.042	0.126	0.74
NMB + DMMB	0.101	0.071	0.034	0.033	0.80
TBO + DMMB	0.381	0.080	0.063	0.020	0.42

## Data Availability

The data used to support the findings of this study are included within the article.
